# From decolonizing global health to neo-colonization by local elites: From the frying pan into the fire

**DOI:** 10.1371/journal.pgph.0004196

**Published:** 2025-02-05

**Authors:** Siddhesh Zadey, Dhananjaya Sharma

**Affiliations:** 1 Association for Socially Applicable Research (ASAR), Pune, Maharashtra, India; 2 NSCB Government Medical College Jabalpur (MP), Jabalpur, India; PLOS: Public Library of Science, UNITED STATES OF AMERICA

## Introduction

In the last two decades, decolonizing global health (DGH) has emerged as a unifying movement in global public health. Paired with the vision of achieving ‘global health equity’ for all [1], the message of decolonization has resonated and grown louder in conferences, journals, and academic circles. Practical guidance is available for the Global South researchers, educators, and practitioners on how to do so [2–5]; yet there is often a disconnection between the rhetoric and reality.

The shift from talk to action has been sluggish, often leaving the power structures intact. Two of the most visible examples are continued domination of scholarly works on DGH by voices from high-income countries and choosing a venue for a conference where getting a visa for scholars from developing countries is a daunting task [6,7]. DGH aims to challenge such *Elite capture* by the Global North funding agencies and academic institutions and even highjacking of health issues such as nutrition and wellness by political leaders and celebrities on social media [8,9].

Here, we would like to focus on the problem of *local* elite capture in the Global South.

## From the frying pan into the fire

Local elites – individuals and institutions that hold majority power in decision-making and enjoy high status within their professional communities – can monopolize opportunities for research, leadership, and funding. This form of *neo-colonization* can prevent broader representation, leading to within-country/community disparities in power and skew narratives that do not strictly adhere to the ‘equity’ goal espoused by Global Health. The metaphor “from the frying pan into the fire” perfectly encapsulates the risk of moving from external colonization to internal neo-colonization. Such *elite swap* - transferring power from the Global North to the select few in the Global South – is in direct contrast to aims of DGH.

A ‘hypothetical’ yet illustrative scenario, drawn from our extensive experience as Global South health professionals, and one that will undoubtedly resonate with our peers, is creation of a prestigious urban research center in a newly independent country which draws Global Health funders and attracts top researchers thus elevating its status and influence. Over time, these local elites gain social capital and power, ensuring that their trainees continue to benefit, leaving smaller institutions and individuals to compete for limited resources. Such local elitism can manifest even in the absence of intentions to create disparities. It also mirrors existing social and gender disparities, with higher-caste, higher-class, and dominant cultural group members more likely to emerge as the local elites. It creates an inefficient hierarchy where privileged individuals from elite institutions, often receive more opportunities without having to show the necessary merit, fostering complacency at the top and discouraging those lower down in the hierarchy who are left struggling for the leftovers that may trickle down from the top. The local elites, positioned at the top of the food chain, often engage in gatekeeping, preventing the trickle-down of resources and opportunities from the Global North that are intended to benefit *all* institutions and individuals. By positioning themselves as representatives of the Global South, without disclosing that they are not true representative of the majority, they evade scrutiny and perpetuate a form of neo-colonialism, allowing them to maintain power both domestically and internationally. Local elite capture is also linked to domestic “helicopter research” and monopolizing ‘task-forces’ meant to address grassroots problems [10,11]. This perpetuates a feudal structure, where local elites in the Global South, in collaboration with their counterparts in the Global North, retain control over power and resources, deepening systemic inequalities.

## Solutions

Preventing the elite swap and mitigating the ongoing local elite capture in the Global South would require democratizing power, diversifying representation, and self-reflection on the part of *all* interest-holders of the DGH movement.

### Democratizing power

It has to start with challenging the *status quo* of representation at global events and questioning why certain familiar faces dominate. We have to ask: how can same familiar faces keep representing six billion people in most Global Health events? Why does that happen? Should that happen? How will that help achieve Global Health equity?

Additionally, Global North donors and investigators must adopt a more inclusive approach, reforming resource allocation to support a ‘culture of excellence’ instead of a ‘centre of excellence’ and trust in institutions, particularly in lower-tier institutions, while engaging more deeply with local communities to identify authentic partners. Such conservatism may stem from concerns about corruption and inefficiency [12]; however, maintaining a presumption of guilt, undermines the mission of equity in global health. There is a need to devise better ways to ensure accountability, transparency, and productivity than to leave out most organizations and perennially invest only in the elite few. And finally, fostering *free and open to all* platforms like SURGhub can help in preventing gatekeeping, decentralizing education and training, breaking the monopoly of elite institutions and addressing historical imbalances in global health [13].

### Diversifying representation

This calls for recognizing the limited role of local elites and involving grassroots leaders, community health workers, and marginalized groups in decision-making - across gender, socio-economic groups, and geography. For instance, in Uganda, the Ministry of Health has actively involved community health workers in shaping public health interventions, which has resulted in more locally relevant and sustainable solutions [14]. Diversifying representation also demands that Global North stakeholders consider complexity of the local socio-cultural hierarchies rather than impose their own diversity models, ensuring that diversity efforts are aligned with the progressive social movements and realities of the Global South.

### Self-reflection

For meaningful democratization and diversification, all stakeholders, including ourselves, must engage in self-reflection about who they truly represent, whether they have done enough to de-elitize global health, and how their actions may inadvertently perpetuate inequities. Most of us see ourselves as leaders or pioneers but do not recognize that we may be acting, inadvertently – without ill intentions - as neo-colonializers in certain contexts. Such self-reflection is not easy and perhaps never complete as there is always more to do. As authors of this paper, we are asking these uncomfortable questions to ourselves daily.

## Conclusion

The decolonization of global health is at a crossroads as the DGH movement risks falling into a trap where local elites replace foreign powers and the underlying power imbalances and inequities remain. To avoid moving from the frying pan into the fire, global health leaders in the Global South must take seriously the need to democratize power, diversify representation, and critically self-reflect on our limitations and methods to achieve the noble intentions of DGH. Decolonization must be more than just rhetoric; it must be about structural change that genuinely empowers all, and not create better opportunities just for a privileged few. This vision of decolonization can only be realized if both external and internal forms of domination in the Global South are successfully challenged. The fear about Global Health expressed earlier ‘*more of the same with some cosmetic changes to disguise supremacy*’ has come back to haunt us [15]. Until there is self-reflective de-elitization, there is no *real* decolonization.
